# EHMTI-0132. Premonitory symptoms and migraine disability assessment (MIDAS) questionnaire

**DOI:** 10.1186/1129-2377-15-S1-D60

**Published:** 2014-09-18

**Authors:** S Sretenovic, A Stanic, A Mitrovic

**Affiliations:** 1 Headache and Migraine Center „Migraine Center", University Clinical Center KBC "Zvezdara", Belgrade, Serbia

## Introduction

Premonitory symptoms are symptoms which precede the migraine attack by 2–48h. Little attention has been paid to the variety of PS and how severely PS are affecting a patient's life.

## Aim

Determine frequency of PS and investigation of a connection between PS and MIDAS scores.

## Methodology

Unsponsored, prospective study lasted for 8 months. In order to be included in the study, subjects had to be above 18 years old, and meet IHS criteria for migraine (without or with aura). Patients have filled in a questionnaire, containing a total of 11 symptoms that have been described as possible migraine PS. Also they have done MIDAS test as a general measure of migraine severity.

## Results

A total of 200 migraine patients (F:M=169:31), mean age 40.6 years (range19-71) participated in this survey. 145 were diagnosed as having migraine without aura and 55 migraine with aura. 69 had a MIDAS score ≤10, 54 between 11 and 20 and 77 scored ≥21. 73 patients took different antimigraine preventive treatments. Most frequent individual premonitory symptoms were fatigue(62.5%) and unhappiness(62.0%) followed by stiff neck(58.5%), photophobia(57.0%), phonophobia(54.5%), concentracion difficulties(50.5%), nausea(48.5%) and osmophobia(45.0%), food craving(35.5%), yawning(34.5%) and water craving(30.0%). Premonitory symptoms fatigue and stiff neck were related with higher MIDAS scores(r²= .041,F(4,4249),p<.001).

## Conclusion

Gender and age did not influence the frequency or the profile of PS. Taking prophylactic therapy for migraine was protective only for nausea. Fatigue and stiff neck were related with MIDAS scores, as they were more frequent, higher were the scores on the test.

No conflict of interest.

